# The metabolism of cells regulates their sensitivity to NK cells depending on p53 status

**DOI:** 10.1038/s41598-022-07281-6

**Published:** 2022-02-25

**Authors:** Sana Belkahla, Joaquin Marco Brualla, Alexis Fayd’herbe de Maudave, Paolo Falvo, Nerea Allende-Vega, Michael Constantinides, Abrar Ul Haq Khan, Lois Coenon, Catherine Alexia, Giulia Mitola, Paul Massa, Stefania Orecchioni, Francesco Bertolini, Wissem Mnif, Javier Hernandez, Alberto Anel, Martin Villalba

**Affiliations:** 1 PYD, King Faisal University, Al Hufuf, Saudi Arabia; 2 IRMB, Univ Montpellier, INSERM, Montpellier, France; 3 Apoptosis, Immunity & Cancer Group, Department Biochemistry and Molecular and Cell Biology, Faculty of Sciences, Campus San Francisco Sq., University of Zaragoza and Aragón Health Research Institute (IIS Aragón), 50009 Zaragoza, Spain; 4 Laboratory of Hematology-Oncology, IRCCS European Institute of Oncology, Milan, Italy; 5 Department of Chemistry, Faculty of Sciences and Arts in Balgarn, University of Bisha, P.O. BOX 199, Bisha, 61922 Saudi Arabia; 6 University of Manouba, ISBST, BVBGR-LR11ES31, Biotechpole Sidi Thabet, 2020 Ariana, Tunisia; 7 IRMB, Univ Montpellier, INSERM, CNRS, CHU Montpellier, Montpellier, France; 8 Institut du Cancer Avignon-Provence Sainte Catherine, 84918 Avignon, France

**Keywords:** Biotechnology, Cancer, Immunology

## Abstract

Leukemic cells proliferate faster than non-transformed counterparts. This requires them to change their metabolism to adapt to their high growth. This change can stress cells and facilitate recognition by immune cells such as cytotoxic lymphocytes, which express the activating receptor Natural Killer G2-D (NKG2D). The tumor suppressor gene p53 regulates cell metabolism, but its role in the expression of metabolism-induced ligands, and subsequent recognition by cytotoxic lymphocytes, is unknown. We show here that dichloroacetate (DCA), which induces oxidative phosphorylation (OXPHOS) in tumor cells, induces the expression of such ligands, e.g. MICA/B, ULBP1 and ICAM-I, by a wtp53-dependent mechanism. Mutant or null p53 have the opposite effect. Conversely, DCA sensitizes only wtp53-expressing cells to cytotoxic lymphocytes, i.e. cytotoxic T lymphocytes and NK cells. In xenograft in vivo models, DCA slows down the growth of tumors with low proliferation. Treatment with DCA, monoclonal antibodies and NK cells also decreased tumors with high proliferation. Treatment of patients with DCA, or a biosimilar drug, could be a clinical option to increase the effectiveness of CAR T cell or allogeneic NK cell therapies.

## Introduction

The main roles of cell metabolism are the conversion of metabolic substrates to energy (catabolism) and the formation of blocks for generating new molecules (anabolism). Tumor cells present a more glycolytic metabolism even in the presence of oxygen. This adaptation is generally known as the Warburg effect^1^, and produces changes in the cells and in their microenvironment^2^. These changes can finally stress cells and induce expression of stress markers, e.g. MHC Class I Polypeptide-Related Sequence A/B (MICA/B) or UL16 Binding Proteins (ULBPs)^3^. The immune system recognizes these signals by the expression of activating receptors in cytotoxic lymphocytes (CL), e.g. the Natural Killer G2-D (NKG2D) activating NK receptor recognizes MICA/B and ULBPs leading to NK cell activation^3^. Therefore, these stress-induced ligands are called NKG2D ligands (NKG2DL^3^). However, tumors generate an immune suppressive environment to avoid destruction by CLs^2,4,5^. This is, at least partially, linked to tumor metabolism that acidifies the tumor microenvironment and inhibits CL function^2,5^. This also jeopardizes the clinical use of allogeneic CL because they are inactivated and/or fail to recognize and/or attack cancer cells in the hostile tumor microenvironment. How overpassing this limitation is one of the major goals of immunotherapy^5,6^. We have recently shown that the antidiabetic drug metformin favors recognition of tumor cells by cytotoxic lymphocytes by increasing expression of NKG2DL and mainly of intercellular adhesion molecule-1 (ICAM-1)^7^. Metformin affects patient’s metabolism by inhibiting gluconeogenesis from redox-dependent substrates such as lactate and glycerol^8^. It can also directly affect cell metabolism by inhibiting mitochondrial complex I, fructose 1,6-bisphosphatase and c-AMP Response Element-binding protein (CREB) and activating AMP-activated protein kinase (AMPK^8^). Therefore, drugs that affect host and/or tumor cell metabolism can regulate the activity of cytotoxic lymphocytes.

Dichloroacetate (DCA) inhibits pyruvate dehydrogenase kinase-1 (PDK1), which phosphorylates and inhibits pyruvate dehydrogenase (PDH)^9^. Hence, DCA treatment leads to PDH activation and pyruvate is mainly diverted to the production of acetyl-CoA. This leads to oxidative phosphorylation (OXPHOS) by activation of mitochondrial complex I^10^. In addition, PDH activation decreases lactic acid production, which is largely responsible of the tumor environment acidification^11^.

DCA induces p53 expression^12^ and its efficacy depends on the status of this tumor suppressor gene. Hence, DCA is more active in cells expressing wt p53^13–16^. p53 is an important metabolic regulator by promoting OXPHOS and inhibiting glycolysis^17^. This involves regulation of several metabolic genes, such as glutaminase 2 (*GLS2*), TP53-induced glycolysis and apoptosis regulator (*TIGAR*), cytochrome c oxidase 2 (*SCO2*), glucose transporter 1 and 4 (*GLUT1* and *GLUT4*)^16,18^. The more common mutations in the *p53* gene abrogate its DNA binding and transactivation activity^19^. p53 mutations are less frequent (10–15%) in hematological malignancies than in solid tumors, however, they associate with chemo-resistance, refractory disease and poor survival^20^. Finally, p53 mutation rate rises in reaction to chemotherapy and in relapse^20^.

Regarding stress ligand expression, p53 seems not to be implicated, at least in mice^21^. In humans, DNA damage-induced NKG2DL upregulation occurs independently of p53^22^. However, human *ULBP1* and *ULBP2* genes contain consensus p53 response elements, and probably p53 action amplifies transcription of certain stress ligands^21^. Moreover, Nutlin-3a, a drug antagonizing the inhibitory interaction of MDM2 with the tumor suppressor p53, induces ULBPs expression in neuroblastoma cells^23^. Regarding the role of p53 on the expression of stress ligands due to metabolic stress, including oxidative stress, is unknown^22,24^. MICA expression in plasma membrane is linked to glycolysis^25^ and to purine nucleotide synthesis^26^. Moreover, metformin, which has strong effect on metabolism, also increases expression of MICA^27^. All these data suggest that metabolic stress can regulate surface expression of stress ligands on tumor cells, recognition by CL and destruction of the “stressed” cell^21^.

Cell-mediated immunotherapy has brought new clinical protocols to cancer, and mainly leukemia, treatment. CTL and NK cells are the main effectors. Although these cells have brought new hope to certain patients, others are insensitive to current treatments^28,29^. The specific tumor metabolism also impacts on their resistance to CL^2,4^. Here, we investigate the effect of reversing tumor metabolism on leukemia cell recognition by CL. We have found that DCA induces on tumor cells the expression of stress ligands and their sensitization to CL.

## Results

### DCA regulates expression of stress ligands in leukemic cells

**Figure 1:**
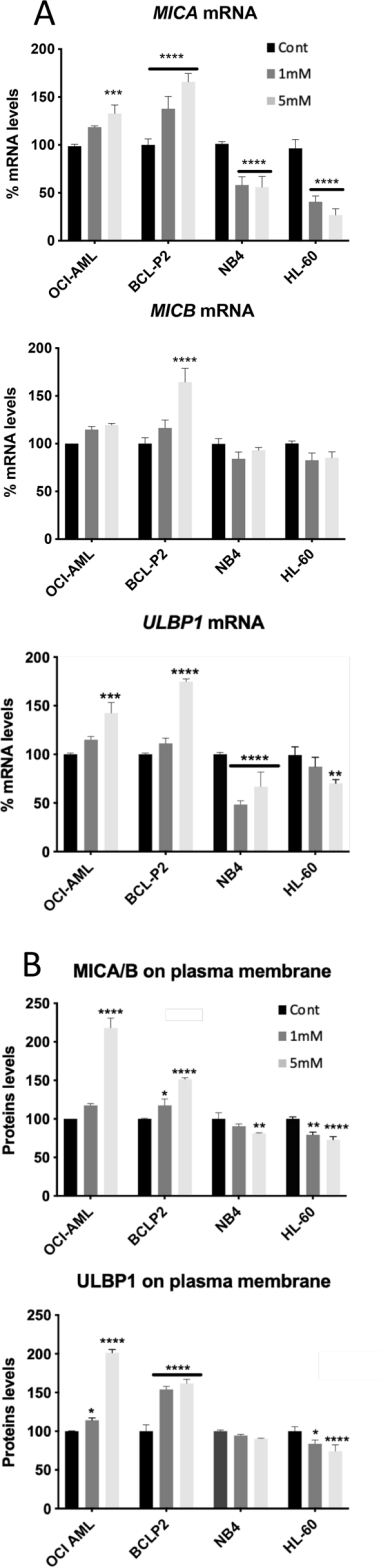
DCA-induced *MICA*, *MICB* and *ULBP1* mRNA expression depends on p53 status in AML cells. (**A**) Different hematopoietic cell lines OCI-AML-3 wtp53, HL-60 nullp53, NB4 mutp53 and a primary cell line BCL-P2 wtp53 were treated with 1 and 5 mM DCA for 1 week and the RNA (**A**) and protein (**B**) levels of the stress ligands were analyzed by qPCR (**A**) and by FACs analysis (**B**). Data represent the percentage of *mRNA* (**A**) or protein (**B**) compared to control, non-treated, cells. The bar graphs represent means ± SD of 3 independent experiments performed in triplicate; *p < 0.05, **p < 0.01, ***p < 0.005, ****p < 0.001, student t-test compare to control cells.

We used DCA concentrations of 1 and 5 mM, which are on the range of those found in plasma of DCA-treated patients that approach 0.5 mM^9,30^. We tested the effect on the expression of 3 representative NKG2DL, i.e. *MICA*, *MICB* and *ULBP1,* in 3 acute myeloid leukemia (AML) cell lines with different p53 status: OCI-AML3 cells express wild type p53 (wt p53), HL60 are p53 null and NB4 express mutant p53 (mut p53) and in a primary cell line that we derived from a B-cell lymphoma patient (BCL-P2) with wt p53^15,16^. DCA increase the mRNA of *MICA* and *ULBP1* on cells expressing wt p53 (Fig. 1A). *MICB* increased or tended to increase especially in BCL-P2 cells. We confirmed these results using an antibody that recognizes MICA and MICB expression on the plasma cell membrane and observed a significant increase in DCA treated cells (Fig. 1B, Supplemental Fig. 1). This was also demonstrated for ULBP1 surface expression. In contrast, in cells lacking wt p53 or having a mutp53, DCA rather decreased expression of *MICA* and *ULBP1* and tended to decrease *MICB* (Fig. 1A). Regarding protein expression, DCA decreased or tended to decrease MICA/B and ULBP1 in these cell lines (Fig. 1B, Supplemental Fig. 1). The effect was dose dependent in all cell lines.

**Figure 2:**
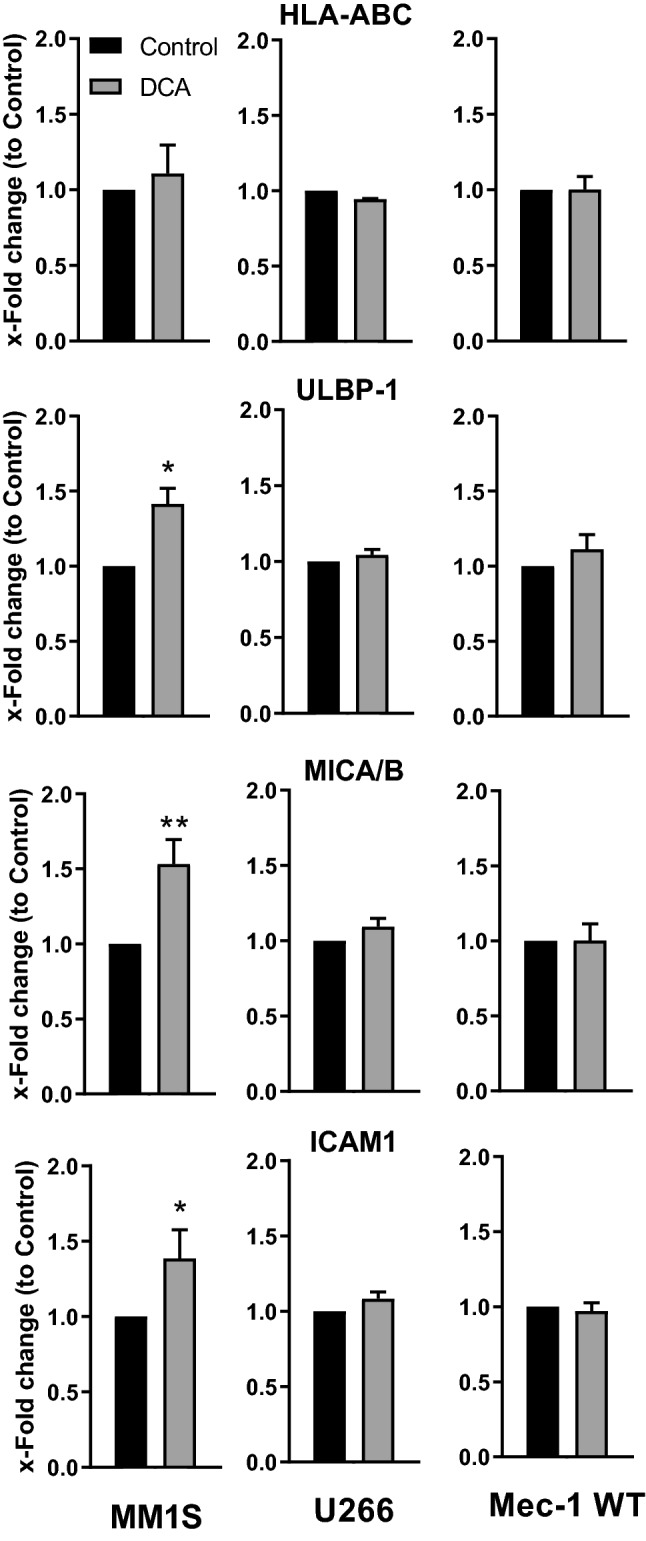
DCA-induced MICA/B, ULBP1 and ICAM-1 protein expression depends on p53 status in MM cells. Different MM cell lines: MM1.S wtp53 and U266 and Mec1 mutp53 were treated with 5 mM DCA for 3 days and expression of the different ligands were examined by FACs analysis. Data represent the relative normalized variation, from MFI values, of treated cells compared to untreated, control, cells. The bar graphs represent means ± SD of 2–5 independent experiments; *p < 0.05, **p < 0.01, ***p < 0.005; Student t-test compare to control cells.

We next used the multiple myeloma (MM) cell lines MM1.S, which expresses wt p53, and U266, which expresses mut p53, and the B-cell chronic lymphocytic leukemia (B-cell CLL) cell line MEC1 (mut p53). DCA increased membrane expression of MICA/B and ULBP1 in MM1.S cells but not in cells expressing mut p53 (Fig. 2). Therefore, these results suggest that stress ligand induction by DCA is dependent on wt p53 expression.

DCA also increased ICAM-1 expression in MM1.S cells, but not in U266 and MEC1 cells, while having no effect on HLA-I expression in any cell line (Fig. 2).

### DCA sensitizes tumor cells to cytotoxic lymphocytes (CL)

**Figure 3:**
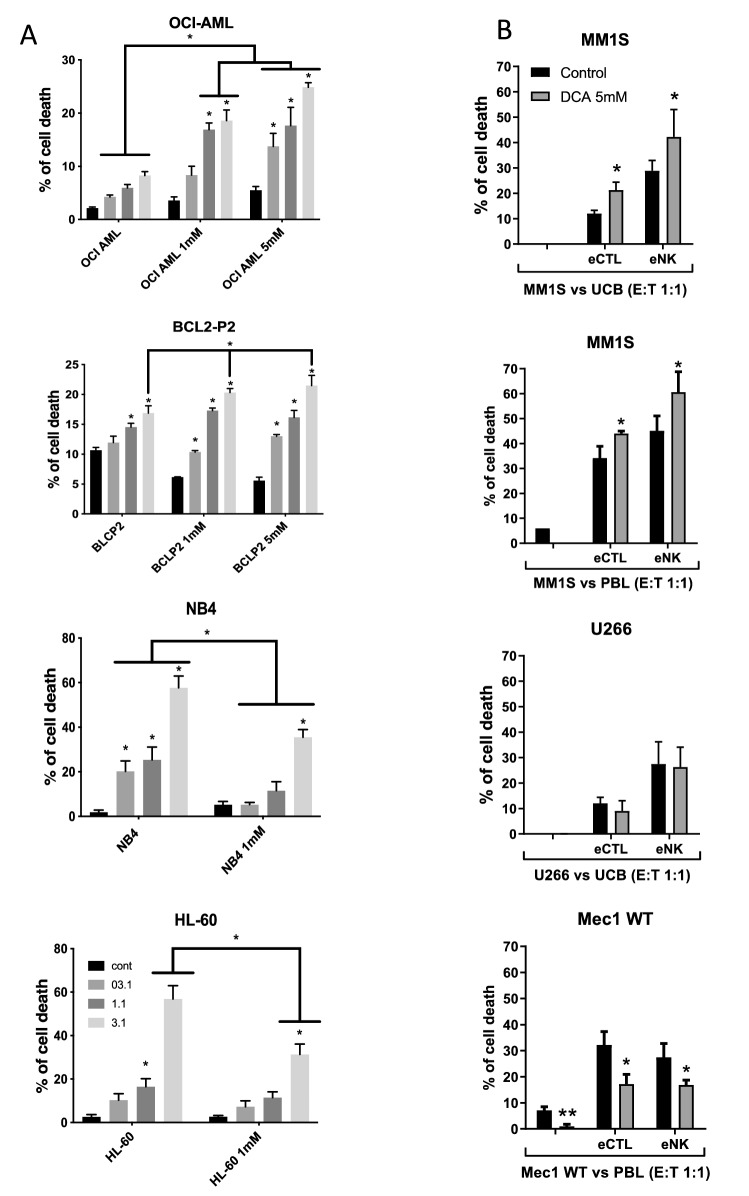
DCA affects NK cell- and CTL-mediated cytotoxicity in leukemic cells depending on tumor p53 status. (**A**) The AML cell lines OCI-AML, NB4 and HL-60 or the primary lymphoma BCL-P2 cells were incubated with 1 mM or 5 mM DCA for 1 week before overnight incubation at different E:T ratios with eNK cells. (**B**) MM cell lines MM1.S and U266 and the lymphoma cell line MEC1 were incubated with 5 mM for 3 days before overnight incubation at E:T ratio 1:1 with e-NK or eCTLs that were produced from UCBs or PBLs. The specific killing of tumor cells was quantified by FACs. The data represent means ± SD; *p < 0.05, **p < 0.01, ***p < 0.001; 2way Anova compared to cells non-treated with eNK or against cells non-treated with DCA as depicted in the graphic.

We next analyzed DCA effect on NK cell-mediated tumor cell killing. For this, we generated expanded and activated NK cells (eNK) from umbilical cord blood (UCB) as previously described^31–33^ and used them against the AML cell lines described in Fig. 1. All cell lines were sensitive to eNK and increasing effector:target (E:T) ratios increased tumor cell killing (Fig. 3A). We have previously shown that at the concentrations used on Fig. 1, DCA is cytostatic, but not cytotoxic, on leukemic cells^12,16^. In agreement with that, DCA alone did not significantly affect survival of the hematopoietic cells used in this study, although it reduced proliferation (Fig. 3, Supplemental Fig. 2). Treatment of the wt p53 cells, i.e. OCI-AML-3 and BCL-P2, with DCA significantly increased eNK-mediated killing at most E:T ratios (Fig. 3A). In contrast, in cell lines lacking wt p53, i.e. NB4 and HL-60, DCA failed to increase eNK-mediated killing (Fig. 3A), and, in fact, it rather decreased it.

We found similar results with the cell lines described in Fig. 2. DCA was not cytotoxic against these cell lines, which were all sensitive to eNK (Fig. 3B). MM1.S were sensitized by DCA to eNK derived from UCB, and also from peripheral blood lymphocytes (PBL) (Fig. 3B). Moreover, because NKG2D is also expressed by CTLs^34,35^, we expanded CTLs (eCTL) from UCB or PBL. DCA also sensitized the MM1.S cells to eCTL (Fig. 3B), but it failed to sensitize U266 and MEC-1 cells, which express mut p53, to eNK or eCTL (Fig. 3B). In the last case, it rather reduced their cytotoxicity, in agreement with the results obtained with the AML cell lines. Altogether, these results indicate that the status of p53 regulates tumor cell sensitivity to CL during metabolic changes.

### The killing mediated by expanded cytotoxic lymphocytes requires LFA-1/ICAM-I interaction

DCA increased expression of stress ligands suggesting that the interaction of NKG2D with these NKG2DL could play a role in DCA-induced sensitization. In contrast, we blocked NKG2D/NKG2DL interaction and we did not observe any effect on DCA-induced eNK or eCTL cytotoxicity in MM1.S cells (Supplemental Fig. 3).

DCA also increased intercellular adhesion molecule-1 (ICAM-1) expression at least in MM1.S cells (Fig. 2). Inhibiting the binding of lymphocyte function-associated antigen-1 (LFA-1) to ICAM-I using an anti-β2 integrin monoclonal antibody (mAb) blocked eNK cytotoxicity and DCA sensitization in the case of eCTL (Supplemental Fig. 3). This is in agreement with previous results showing that ICAM-I/LFA-1 interaction is essential for target cell recognition by CL^36,37^ and correlates with p53-dpendent ICAM-1 induction by DCA.

DCA could also sensitize tumor cells to CL by increasing death receptor (DR) expression because CL express several DR ligands including FasL and TRAIL, which binds to DR4 and 5^38^. DCA increased DR5 but not Fas or DR4 in the wt p53 MM1.S cell line and none of them in MEC1 cells (Supplemental Fig. 4A).

To further investigate this, we used the blocking construct Fas-Fc, which blocks Fas/FasL interaction, and the blocking anti-TRAIL mAb RIK2, which blocks the interaction of TRAIL with DR4 and DR5. Fas-Fc did not have any effect, but RIK2 statistically decreased, but not abolished, eNK-mediated cytotoxicity in resting or DCA-treated cells (Supplemental Fig. 4B). Blocking degranulation with EGTA largely decreased eNK cytotoxicity (Supplemental Fig. 4B). These results suggest that DCA made tumor cells more sensitive to CLs by multiple mechanisms, including stress ligand, ICAM-1 and DR5 upregulation.

### DCA signals stress ligand expression through p53

**Figure 4:**
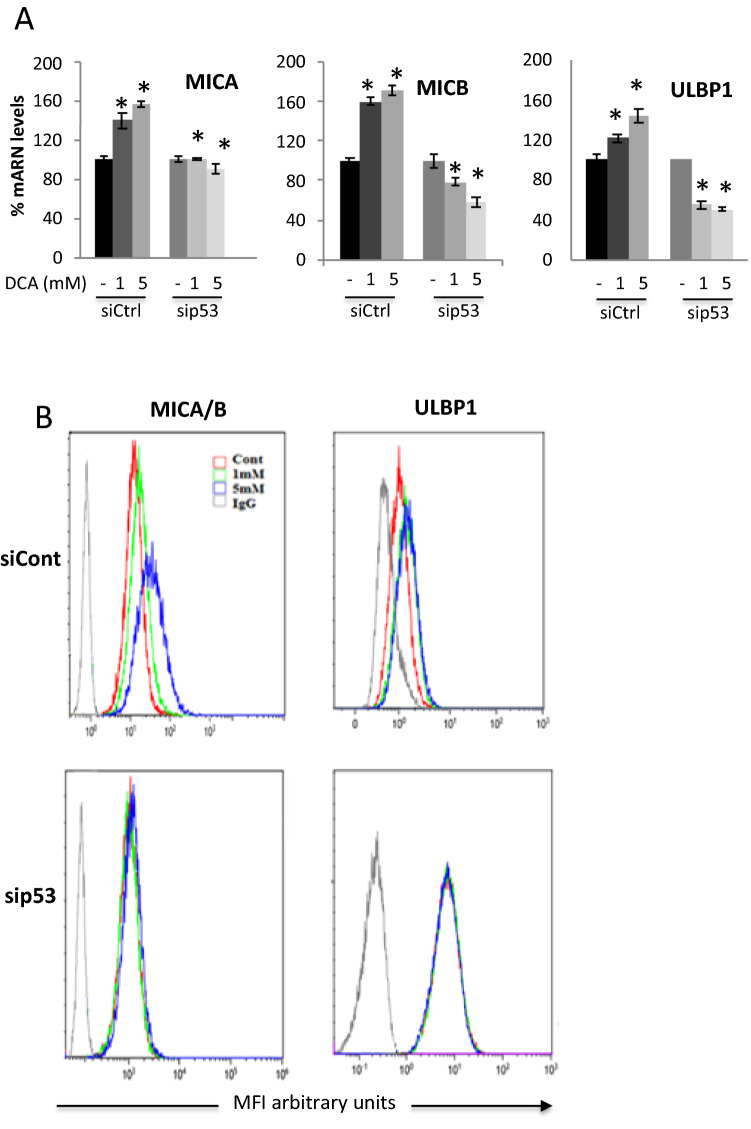
Decreasing p53 levels prevents DCA-induced stress ligands upregulation. (**A**) OCI-AML3 cells were transfected with siRNA for p53 or control siRNA and 24 h later were treated with 1and 5 mM DCA for 3 days. Protein and mRNA levels were quantified as described in Supplemental Fig. 1 and Fig. 1, respectively. Bar graphs denote the % of mRNA compared to non-treated cells and represent means ± SD of 3 independent experiments; *p < 0.05, **p < 0.01, ***p < 0.005 student t-test compare to non-treated cells. (**B**) The expression of stress ligands in cell membrane were analyzed by FACs.

To further investigate the role of p53 in our observations, we transduced a siRNA for p53 (sip53) in OCI-AML3 as previously described^15^ efficiently reducing *p53* mRNA (Supplemental Fig. 5A) and p53 protein (Supplemental Fig. 5B). Cells transfected with a control siRNA (siCtrl) upregulated stress ligand mRNA or protein upon DCA treatment as the non-transfected cells (Fig. 4). In contrast, sip53 expressing cells did not increase stress ligands mRNA after DCA treatment (Fig. 4A). In fact, these cells behave as cell lines lacking wt p53 expression or expressing a mut p53 (Fig. 1), rather reducing stress ligand expression. This correlated with a lack of increase in MICA/B and ULBP1 proteins at the plasma membrane after DCA treatment (Fig. 4B).

### DCA requires p53 to efficiently sensitize target cells to eNK cells

**Figure 5:**
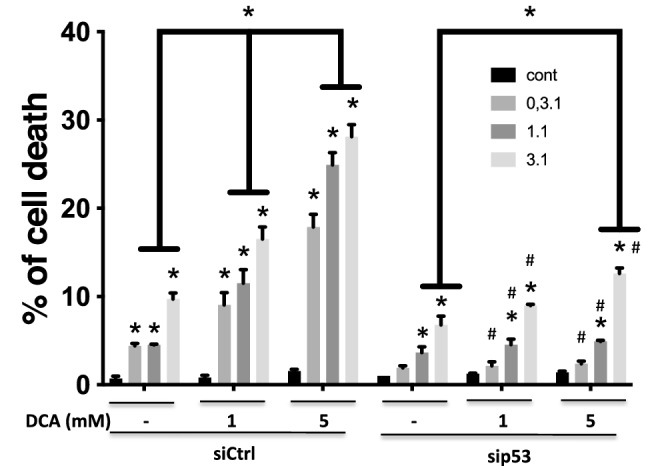
Decreasing p53 levels diminishes the DCA-induced increase in cytotoxicity. OCI-AML3 cells were treated as in Fig. 4 and then incubated overnight with e-NK cells at different E:T ratios. The specific killing of tumor cells was quantified by FACs. The data represent means ± SD of 3 independent experiments; *p < 0.001; 2way Anova compared to cells non-treated with eNK or against cells non-treated with DCA (depicted in the graphic); ^#^p < 0.001; 2 way Anova compared to cells transfected with siCtrl.

Similar to non-transfected cells, OCI-AML3 cells expressing a siCtrl were susceptible to eNK and DCA sensitized them further (Fig. 5). Cell transfected with sip53 tended to be less sensitive to eNK cell cytotoxicity at all E:T ratios assayed. In these cells, DCA sensitization was not observed at 1 mM DCA and at 5 mM at the lowest E:T ratios (Fig. 5). Remarkably, after DCA treatment, cells expressing sip53 were killed at significantly lower percentages than cells expressing siCtrl at both DCA concentrations and at all E:T ratios (Fig. 5).

### DCA delays growth of fast-growing lymphoma cells in vivo in the presence of an anti-CD20 mAb

**Figure 6:**
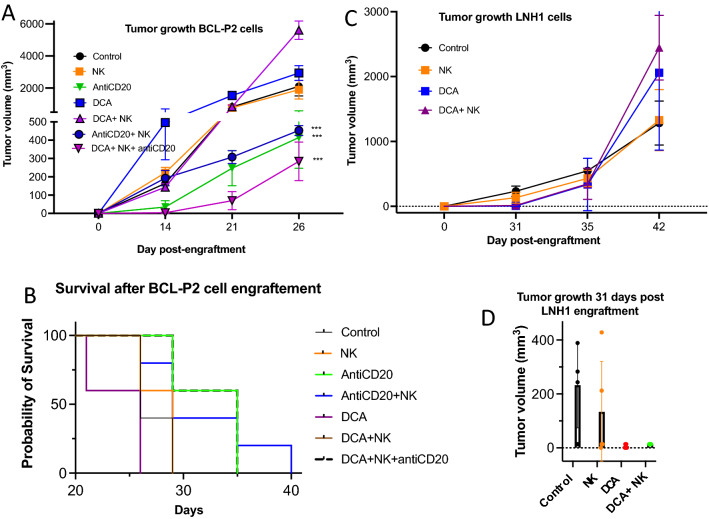
DCA slightly affect cell tumor growth in NSG mice engrafted with lymphoma cells. (**A**) Five NSG mice/group were subcutaneously engrafted with 5 × 10^6^ BCLP2 and treated with DCA, with e-NK and/or with an anti-CD20 mAb. Graphs show tumor cell growth. (**B**) Mice survival after different treatments. (**C**) Five NSG mice/group were subcutaneously engrafted with 10 × 10^6^ LNH1 (right) cells and treated with metformin and/or with e-NK. Graphs show tumor cell growth. (**D**) Tumor cell growth at day 31 after LNH1 engraftment. Graphs show means ± SD, ***p < 0.005; 2 way Anova compared to non-treated cells.

We next investigated the effect of DCA in combination with eNK cells on lymphoma cell development in vivo. We used the BCL-P2 lymphoma that quickly induce tumors when engrafted subcutaneously at 5 million cells per mice in NSG mice^31^. DCA or eNK did not affect tumor development when used in monotherapy or in combination (Fig. 6A). Consequently, mice survival was not increased by these treatments (Fig. 6B). However, tumor development was efficiently delayed by the anti-CD20 mAb rituximab, which also increased mice survival (Fig. 6A,B). Combination of the anti-CD20 mAb with eNK cells did not increase further the effect of rituximab alone. In contrast, the combinatory treatment of DCA, eNK and anti-CD20 mAb further decreased tumor development at all times tested.

### DCA delays growth of a slow-growing lymphoma in vivo

We next investigated DCA effect on the grow of LNH1 cells, which are issued from a diffuse large B-cell lymphoma (DLBCL) patient. These cells grow slower than BCLP2 when subcutaneously engrafted at 10 million cells per mice^31^. eNK failed to affect tumor growth (Fig. 6C). DCA delayed tumor grow at shorter times after engraftment (Fig. 6D), but eNK engraftment did not have any additional effect (Fig. 6C). Of note, DCA was given to mice only during the first ten days after tumor engraftment. The antiCD20 mAb totally abrogated the growth of these cells (data not shown), making irrelevant the cotreatment with DCA and/or eNK.

## Discussion

Treatment of highly resistant tumor cells in refractory patients requires the development of new therapies or the combination of current ones. We have already described the increased sensitivity of wtp53-expressing cells to the combination of chemotherapeutic and metabolic drugs^12,15,16^. The use of metabolic drugs that sensitize tumor cells to CL is another interesting possibility^2,4,6,7^. Our results show that the tumor p53 status relates to the effectivity of cotreatments involving these therapies. This is also a limitation of the combinatory therapy, i.e. metabolic drugs and allogeneic CL, that we propose here. It should be noted that the p53 status is routinely controlled in cancer patients in most hospitals. This could allow clinicians to choose the most appropriate treatment involving metabolic drugs and/or CL. If patient express a wtp53, it would be interesting to pretreat patients with drugs that favor an OXPHOS metabolism before engraftment with CL such as NK and T cells. We have used here DCA concentrations similar to that used in patients^30,39^ and DCA is relatively well-tolerated^30,39^, supporting its use or another similarly acting drug. CD8 T cells express NKG2D^40^, hence DCA sensitization could also include CAR T cell therapy. A similar concept has been proposed in neuroblastoma by using Nutlin-3a, which restores p53 activity and leads to expression of ligands for NKG2D and DNAM-1^23^.

A direct up-regulation of NKG2DL by wt p53 has been described only for *ULBP1* and *2* upon binding of overexpressed p53 to response elements located in their promoters^21,22^. We show here that p53 status mediates NKG2DL expression during metabolic stress induced by DCA. However, we believe that the effect is not direct and that wtp53 is rather involved in favoring the metabolic changes that finally lead to stress ligands expression. The exact mechanism underlining this expression is unknown, but recent findings support the following hypothesis. Tumor metabolism is not static, it changes during tumor evolution and accordingly to changes in tumor micro- and macro-environment^2,41,42^. Hence in certain situations tumor cells change their metabolism to take advantage of the available resources. Perhaps the best example is the increase in fatty acid (FA) consumption during metastasis^43^. Interestingly, metastatic cells are better recognized by NK cells than primary tumor cells^44–46^, although the exact mechanism is unknown. Short-chain fatty acids (SCFAs) increase the availability of acetyl-CoA and acetylation of unknown targets, which induce MICA/B expression^24^. This requires the phosphoenolpyruvate carboxykinase (PEPCK), the rate-limiting enzyme in gluconeogenesis^24^. Interestingly, DCA by inhibiting PDK1 and activating PDH also leads to accumulation of acetyl-CoA^47^ and inhibits gluconeogenesis^48^. Moreover, DCA affects lipid metabolism by increasing lipid intake by cells^49^. Hence, our results suggest that the DCA-mediated increase on lipid metabolism could underline DCA-induced NK cell sensitization. In this sense, p53 modulates lipid metabolism and it is generally accepted that wtp53 positively regulates oxidative phosphorylation and lipid catabolism whereas negatively regulates lipid synthesis and glycolysis^50,51^. mtp53 in tumor cells does the opposite, positively regulating lipid synthesis and glycolysis^50,51^. Therefore, we propose that DCA activates wtp53 activity^12–16^ leading to increase lipid catabolism^49^, increasing availability of acetyl-CoA and acetylation of unknown targets^24^ and NKG2DL expression^24^. However, p53 is a pleiotropic gene that affects multiple cell functions, e.g. apoptosis, metabolism, etc. Hence, this and/or other mechanisms could underline p53-induced NKG2DL expression under metabolic changes.

Wt p53 activation by DCA can also be implicated in the observed ICAM-1 and DR5 up-regulation. Blocking TRAIL interaction with its receptors with the blocking mAb RIK2 clearly reduces DCA-induced sensitization. In addition, blocking ICAM-1 interaction with LFA-1 decreases eNK-mediated killing and DCA-induced sensitization to eCTL.

It has been described that ROS generation, through NF-κB activation, and also p53, contribute to ICAM-1 upregulation in different cellular settings^52–54^. DCA increases ROS generation and also results in p53 activation^12,16^. This could lead to ICAM-1 induction and facilitate the effective binding of CL^55^ and gives stimulating signals that favor target elimination^55^. In addition, it has also been demonstrated that p53 transactivates DR5 gene expression^56^, explaining p53-dependent DR5 up-regulation by DCA and their sensitization to CL. In fact, we demonstrate that p53 down-regulation by genetic manipulation in tumor cells greatly reduces eNK cytotoxicity against them, and also DCA-mediated sensitization.

In summary, our previous results^7^ and those presented here show that changes in metabolism can sensitize tumor cell to CL by different mechanisms and ICAM-1 overexpression looks the more relevant. However, we have used activated NK cells. Naïve NK cells could better sense NKG2DL upregulation on tumor targets than our eNK. If naïve or activated NK cells are more representative of the situation in the tumor microenvironment is unclear. Once in the tumor microenvironment NK cells should be activated by the targets and/or the pro-inflammatory cytokines. In contrast, for tumor cells outside of this environment, e.g. metastatic cells, it could be the opposite.

Regarding in vivo experiments. we treated mice with DCA only during the first 10 days after tumor engraftment. Hence, after this initial treatment, tumor cell progress in a DCA-free environment. We observed that DCA delayed tumor growth in the slow growing LNH1 model, and NK cells did not improve its effect. In the fast growing BCL-P2 lymphoma DCA improves survival and decreases tumor growth in combination with an effective anti-CD20 mAb and with eNK cells. In our mouse models, eNK are in contact with DCA during their antitumor function, which is not the case of the in vitro experiments. It is well-known that CL require a glycolytic metabolism for their maximal activity and educated NK cells display a high glycolytic rate^57^, which is essential for their antitumor function^58^. Hence, DCA could affect in vivo eNK metabolism and impair their function. The anti-CD20 mAb could overpass this effect by inducing maximal NK cell activation through potent antibody-dependent cell mediated cytotoxicity (ADCC) mediated by CD16 receptor, which recognizes the Fc moiety of IgGs. On the other hand, lactate acidosis is eliminated by DCA, favoring immune cell-mediated tumor elimination in vivo^11^. Hence, the overall in vivo DCA effect on anti-tumor immune surveillance is multifactorial and not easy to predict.

The antibody–drug conjugates (ADCs) are gaining interest in oncology. Antibodies carrying cytotoxic agents target and deliver the drug to the tumor cell and/or the tumor site^59^. An interesting possibility to avoid the possible DCA-induced widespread damage or widespread metabolic changes would be to conjugate DCA to an antibody directed against an antigen preferentially expressed by tumor cells.

Regarding the clinical implications of our findings, DCA has been largely used in medicine to treat metabolic disorders^9,39^ and has shown clinical efficacy for treatment of certain cancers^30,60,61^. However, the mechanisms responsible for DCA-induced tumor regression have not been clearly elucidated and in fact DCA is effective in a very limited number of cancer patients. Our results suggest that this drug, or a biosimilar, could facilitate tumor cell recognition by CL. We believe that a short, but intensive, DCA treatment previous to allogeneic CL, i.e. NK, CAR T cells…, infusion could favor cancer cell recognition and patient’s prognosis.

In summary our current results clearly show that tumor metabolism affects the recognition by CL and hence, the immunotherapy mediated by these effectors.

## Materials and methods

### Ethical statement

Experiments involving animals were approved by the Italian National Institute of Health the 08/29/2019 under the number 639/2019 and have been done in accordance with the applicable Italian laws (D.L.vo 26/14 and following amendments), with the Institutional Animal Care and Use Committee, and with the institutional guidelines at the European Institute of Oncology. Our study is reported in accordance with ARRIVE guidelines (https://arriveguidelines.org).

The use of human specimens for scientific purposes was approved by the French National Ethics Committee. All methods were carried out in accordance with the approved guidelines and regulations of this committee. Written informed consent was obtained from each patient or donor prior to sampling.

### Human samples

Data and samples from patients were collected at the Clinical Hematology Department of the CHU Montpellier, France, after patient’s written consent and following French regulations. Patients were enrolled in the HEMODIAG_2020 (ID-RCB: 2011-A00924-37) clinical program approved by the “Comités de Protection des Personnes Sud Méditerranée I” with the reference 1324. Samples were collected at diagnosis and kept by the CHU Montpellier^16,31,62^. Peripheral blood mononuclear cells (PBMCs) were obtained by ficoll gradient and stored frozen in liquid nitrogen until use.

### Cell lines and culture conditions

The leukemic human cell lines OCI-AML3, NB4, HL-60, MM1.S, U266 and MEC1 were grown in RPMI 1640–Glutamax (GIBCO) supplemented with 10% FBS as previously described^63,64^. All these cell lines were originally obtained from the ATCC (https://www.atcc.org/cell-products#t=productTab&numberOfResults=12). Their p53 status was have been previously described^12,15,16^. We confirmed the tumor p53 statusbysequencing the entire open reading frame by Eurofins MWG Operon with the primers E67F (5′-TTGCGTGTGGAGTATTTGGAT-3′) and MP9ER (5′-TCTCCCAGGACAGCACAAACACG-3′)^16^. The primary cell line from a lymphoma B cell patient (BCL-P2) were grown in the same medium with 10% FBS.

### Reagents and antibodies

DCA was from Aldrich. Fluorescence coupled antibodies against human ULBP1-488 and the blocking antibody against NKG2D (MAB139) were from R&D Systems; ICAM-1, MICA/B, CD155, CD33, CD138 and HLA were from Miltenyi; DR4 (12-6644-73), DR5 (12-9908-42) and Fas (BMS140FI) were from eBiosciences and 7AAD from BD Biosciences. Recombinant human Fas-Fc chimera (AFA0114121) was purchased from R&D Systems and human TRAIL-blocking antibody RIK2 (550912) from BD Biosciences. For in vivo experiments we used the anti-CD20 rituximab. The D1D2 construct that binds to LFA-1 has been described^31^. Blocking anti-ULBP1 antibody MAB1380, anti-MICA/B antibody MAB13001 and anti-β2 integrin AF1730 were from R&D systems.

### NK cell isolation and expansion

This work benefited from umbilical cord blood units (UCBs) and the expertise of Prof. John De Vos, in charge of the Biological Resource Center Collection of the University Hospital of Montpellier—http://www.chu-montpellier.fr/en/platforms (BIOBANQUES Identifier—BB-0033-00031). NK cells were expanded as previously described^31,32^. Briefly, UCBMCs or PBMCs were isolated through density gradient centrifugation using Histopaque-1077 (Sigma). Blood samples were diluted at 1:1 ratio with RPMI then layered above 10 mL Histopaque in a 50 mL conical tube. Once centrifuged for 30 min at 400×*g*, the white layers of mononuclear cells (MCs) were collected and washed. Using EasySepTM Human CD3 Positive Isolation kit (StemCell Technologies), the CD3^+^ cell fraction (T and NKT cells) of the MCs was depleted in each sample to better culture the NK cells. Once depletion was verified through flow cytometry, cells were cultured for 20 days. NKs were cultured with γ-irradiated PLH at a 1:4 (NK cell: feeder cell) ratio and IL-2 (100 IU/mL) and IL-15 (5 ng/mL). Feeder cells and cytokines were refreshed every 3–4 days. To monitor expansion, NK cells were stained with APC-labelled anti-CD3 mAb and PE or Vio770-labelled anti-CD56 mAb (both form BD Biosciences). Culture viability was determined at regular intervals through flow cytometry analysis.

### eCTL expansion

All eCTLs used were generated from PBMCs of healthy donors obtained from leukopaks provided by the Blood and Tissue Bank of Aragón, under the permission of the Clinical Research Ethical Committee from Aragón (CEICA) (Ref. PI16/0129). Briefly, PBMCs were isolated through density gradient centrifugation using Histopaque-1077 (Sigma), as stated above. Afterwards, PBMCs were cultured for 20 days with PLH (previously inactivated with mitomycin C at 25 µg/mL) at a 1:1 (PBMC:feeder cell) ratio and IL-2 (100 IU/mL) and IL-15 (5 ng/mL). Feeder cells and cytokines were refreshed every 3–4 days. Expansion was monitored by PBMC staining with mAb CD3-FITC or mAb CD56-APC, both from Miltenyi Biotech. Culture viability was determined at regular intervals through flow cytometry analysis. Once obtained eCTL, CD3^+^ population was selected using EasySepTM Human CD3 Positive Isolation kit (StemCell Technologies) and CD3^+^/CD56^-^ presence was confirmed by flow cytometry.

### In vivo experiments

In vivo experiments were carried out using 6 to 8 weeks/old male NOD scid gamma (NSG) mice, which were bred and housed in pathogen-free conditions in the animal facility of the European Institute of Oncology–Italian Foundation for Cancer Research (FIRC), Institute of Molecular Oncology (Milan, Italy). For engraftment of human cells, mice were subcutaneously engrafted with 5 × 10^6^ BCL-P2 or 10 × 10^6^ LNH1 primary tumor cells derived from a BCL (P2) patient or a diffuse large B-cell lymphoma (DLBCL) patient (LNH1). DCA 50 mg/kg was given at one dose gavage at days 1, 2 3, 8, 9 and 10 after engraftment. At day 4, we engrafted 15 (BCL-P2) or 10 (LNH1) million e-NK cells and at day 6, mice were treated i.p. with the anti-CD20 rituximab (in saline medium) 3 mg/kg once a week × 3 weeks; or with a combination of both. Tumor growth was monitored at least once a week using a digital calliper, and tumor volume was calculated according to the formula: L × W^2^/2 = mm^3^, where W represents the width and L the length of the tumor mass.

### Transient transfection

Control and p53 siRNA were ON-TARGETplus SMARTpools (mixture of 4 siRNA) from Dharmacon. OCI-AML-3 cells were transfected using Amaxa ™ D-Nucleofector ™ Lonza Kit according to manufactured protocol.

### Counting and determination of cell viability

After treatment, hematopoietic cells were stained with Muse^®^ Count and Viability Reagent, and then analyzed on the Muse^®^ Cell Analyzer (Millipore) to identify cell number and survival^12^.

### NK cell mediated cytotoxicity

Fresh or frozen (stored in liquid nitrogen) NK cells were labeled with 3 µM of CellTracker™ Violet BMQC Dye (Life Technologies) and incubated overnight with target cells at different E:T ratios. Subsequently, viability was analyzed in the violet fluorescence negative target cell population by flow cytometry using 7AAD (BD Biosciencies)^31^.

For certain experiments, effector cells were previously incubated with anti-TRAIL mAb RIK2 (2.5 µg/mL), recombinant human Fas-Fc chimera (10 ng/mL) or calcium chelator EGTA (1 mM) for 1 h at 37 °C, before facing target cells.

### RT-PCR and DNA sequencing

Total RNA was extracted using NucleoSpin RNA isolation columns (Macherey–Nagel), reverse transcription was carried out using iScript™ cDNA Synthesis Kit (Biorad). Quantitative PCR was performed with KAPA SYBR Green qPCR SuperMix (Cliniscience) and a CFX Connect™ Real-Time qPCR machine (Biorad) with MICA, MICB, ULBP1 and actin primers. All samples were normalized to β-actin mRNA levels. Results are expressed relative to control values arbitrarily set at 100. The primers used were:

- *MICA* Forward: (5′-GAACGGAAAGGACCTCAGGA-3′) Reverse: (5′-ATATTCCAGGGATAGAAGCCAGAA-3′),
- *MICB* Forward: (5′-CTCGTGAGTCCAGGGATCTA-3′) Reverse: (5′-GCGTTTCTGCCTGTCATAGC-3′),
- *ULBP1* Forward: (5′-GATCCAACAAAACCACCCTCTCT-3′) Reverse: (5′-ACTTTCCACCTGTCACTCTAAACAAC-3′),
- *Actin* Forward: (5′-GAGGGAAATCGTGCGTGACA-3′) Reverse: (5′-AATAGTGATGACCTGGCCGT-3′).

### Flow cytometry

Briefly, 1 × 10^6^ cells were stained with antibody in PBS with 2% FBS and incubated at 37 °C for 30 min. Cells were then washed and suspended in 200–250 μL PBS 2% FBS with the corresponding antibodies. Staining was analyzed using a Gallios flow cytometer (Beckman) and Kaluza software.

### Statistical analysis

The statistical analysis of the difference between means was performed using the 2way ANOVA test or the Student’s test using the software Prism9 from GraphPad Software, LLC. The results are given as the confidence interval (*p < 0.05, **p < 0.01, ***p < 0.005). All the experiments described in the figures with a quantitative analysis have been performed at least three times in duplicate. Other experiments were performed three times with similar results.

## Supplementary Information

Supplementary Figures.
