# Supplementary material for: The metabolism of cells regulates their sensitivity to NK cells depending on p53 status
Source: Sci Rep. 2022 Feb 25;12:3234. doi: 10.1038/s41598-022-07281-6 (PMC8881467; doi:10.1038/s41598-022-07281-6)
Supplement: Supplementary file 1 — Supplementary Figures. [file 41598_2022_7281_MOESM1_ESM.pdf]

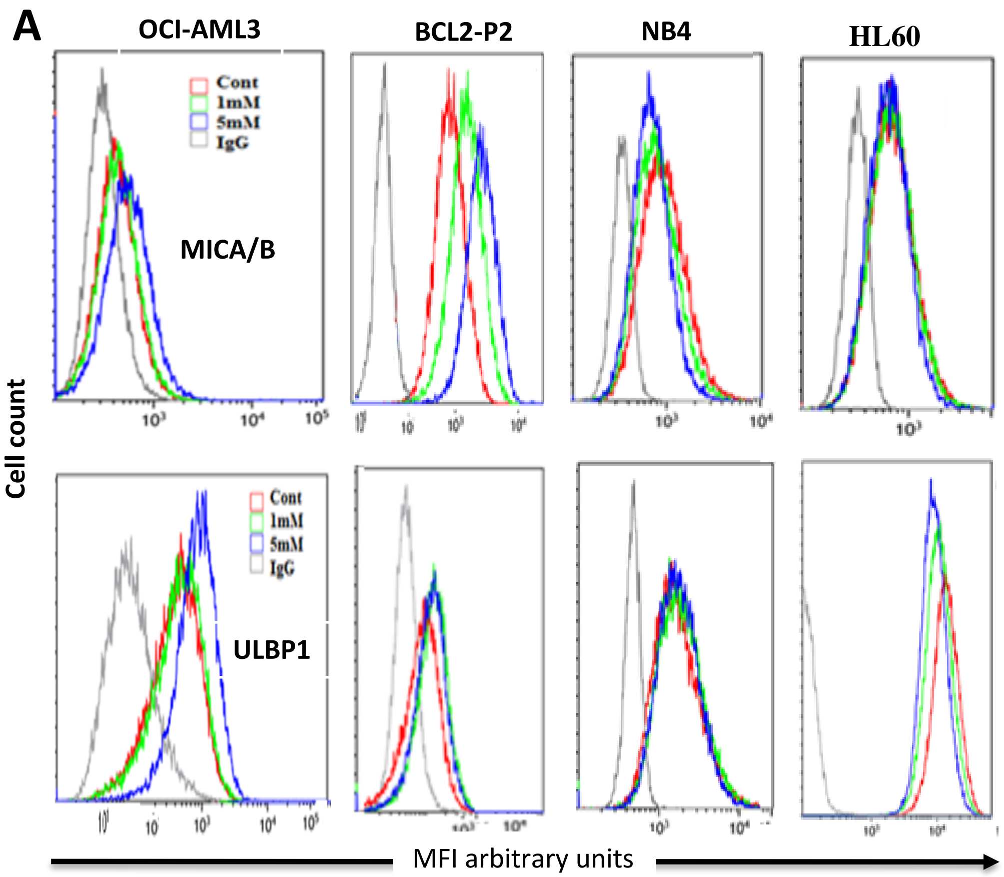

Supplemental Figure 1. DCA-induced MICA, MICB and ULBP1 protein expression depends on p53 status in leukemic cells. Different hematopoietic cells were treated as in Fig. 1 and expression of stress ligands in the surface of the cells were analyzed by FACs. The expression of the stress ligands in the surface of the cells were analyzed by FACs.

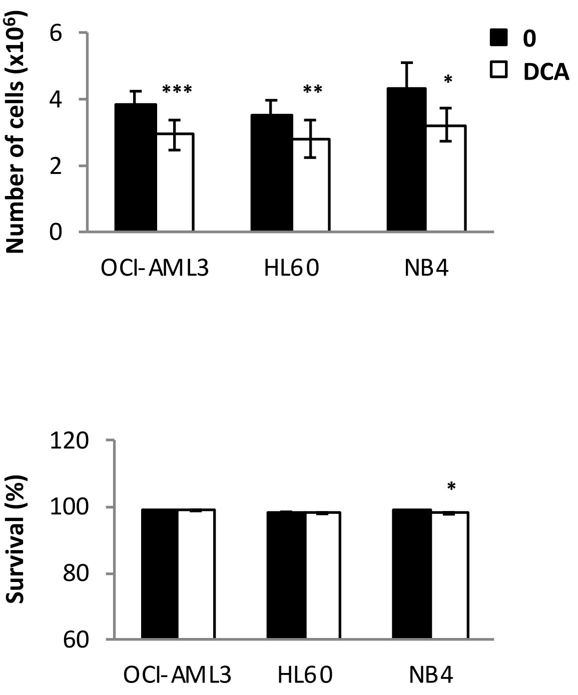

Supplemental Figure 2. DCA is cytostatic, but not cytotoxic, on AML cell lines. Different hematopoietic cell lines OCI-AML-3 wtp53, HL-60 nullp53, NB4 mutp53 were treated with 5mM DCA for 2 days. Cells were stained with Muse® Count and Viability Reagent, and then analyzed on the Muse® Cell Analyzer to identify cell number and survival. The bar graphs represent means ± SEM of 6 independent experiments; * $p<0.05$, ** $p<0.01$, *** $p<0.001$ student t-test compare to control cells.

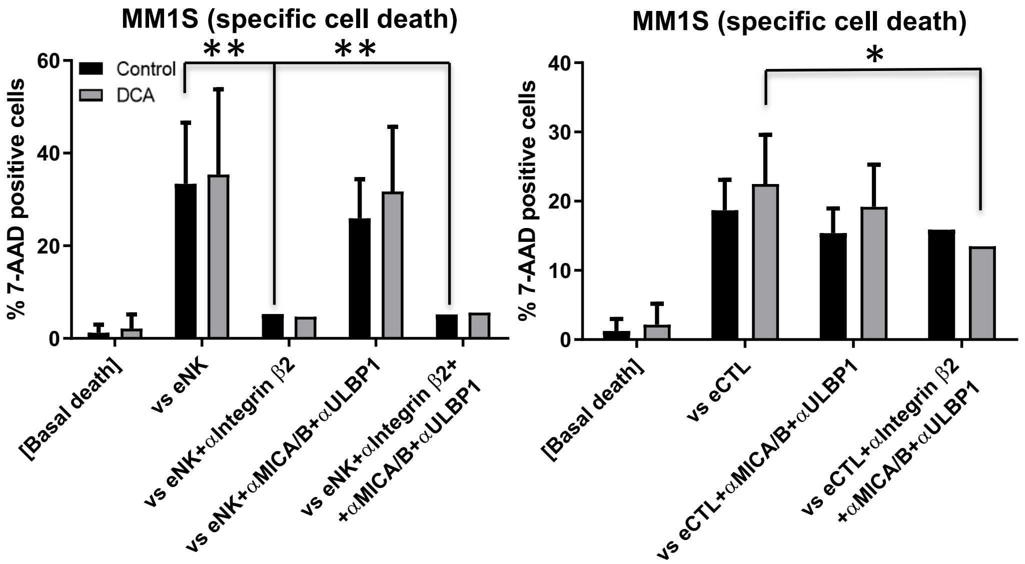

Supplemental Figure 3. LFA-1/ICAM-I interaction regulates cytotoxicity by expanded cytotoxic lymphocytes. MM1.S cells were incubated with 5 mM DCA for 3 days before overnight incubation with blocking molecules (10 µg/ml of α-Integrin β2, α-MICA/B and/or α-ULBP1) and with eNK or eCTLs at E:T ratio 1:1. The specific killing of tumor cells was quantified by FACs. The data represent means ± SD; *p < 0.05, **p < 0.01, ***p < 0.001 Student's t-test compared to control cells or as depicted in the graphic.

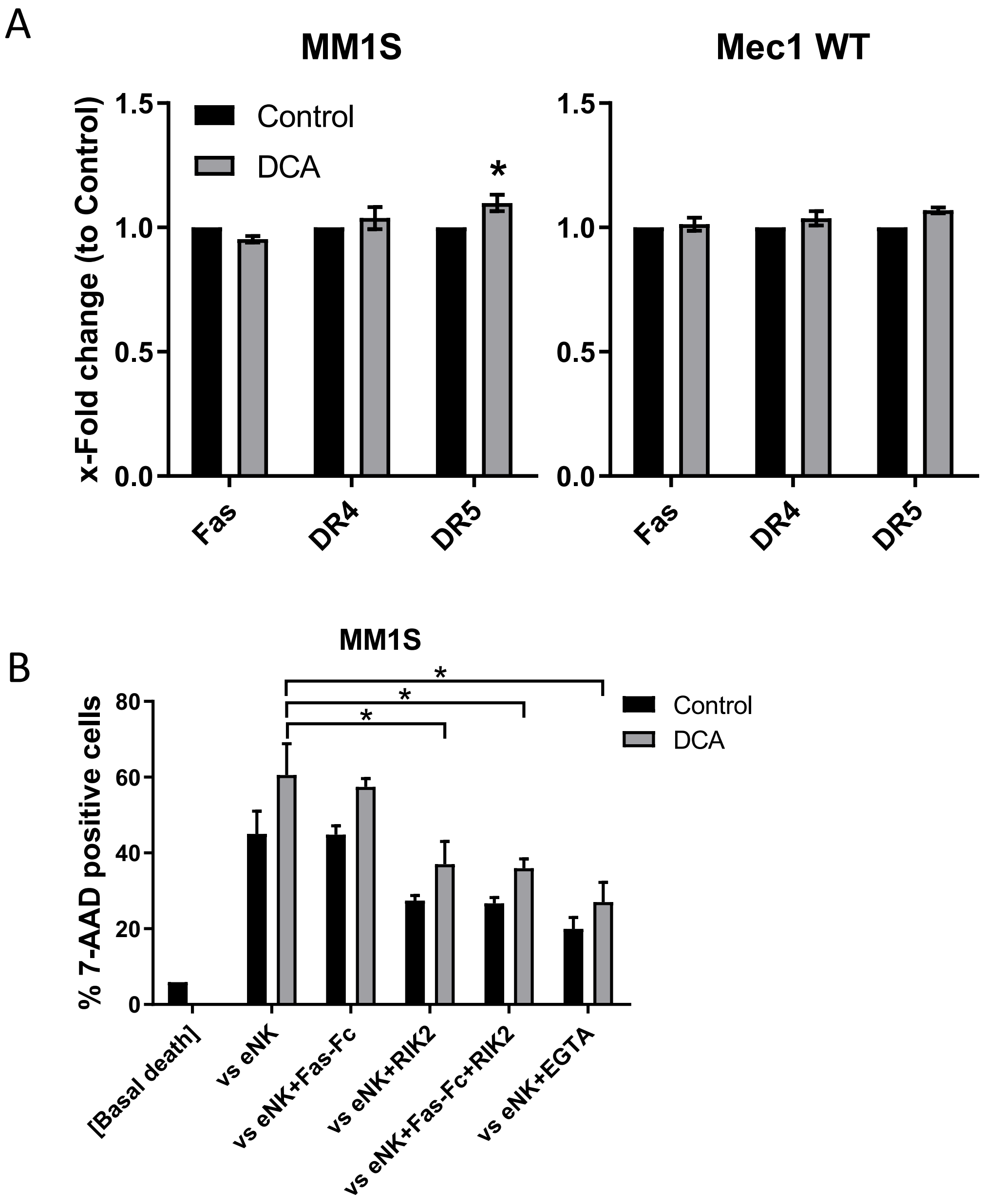

Supplemental Figure 4. eNK partially kill tumor target cells by engagement of death receptors. A) MM1.S wtp53 and Mec1 mutp53 were treated with 5 mM DCA for 3 days and expression of the different ligands were examined by FACs analysis. Data represent normalized MFI values compared to untreated, control, cells. B) MM1.S cells were incubated with 5 mM DCA for 3 days before overnight incubation with blockin molecules (1 mM EGTA, 10 ng/ml Fas-Fc and/or 2,5 µg/ml RIK2) and with eNK at E:T ratio 1:1. The specific killing of tumor cells was quantified by FACs. The data represent means ± SD; *p < 0.05, **p < 0.01, ***p < 0.001 Student's t-test compared to control cells or as depicted in the graphic.

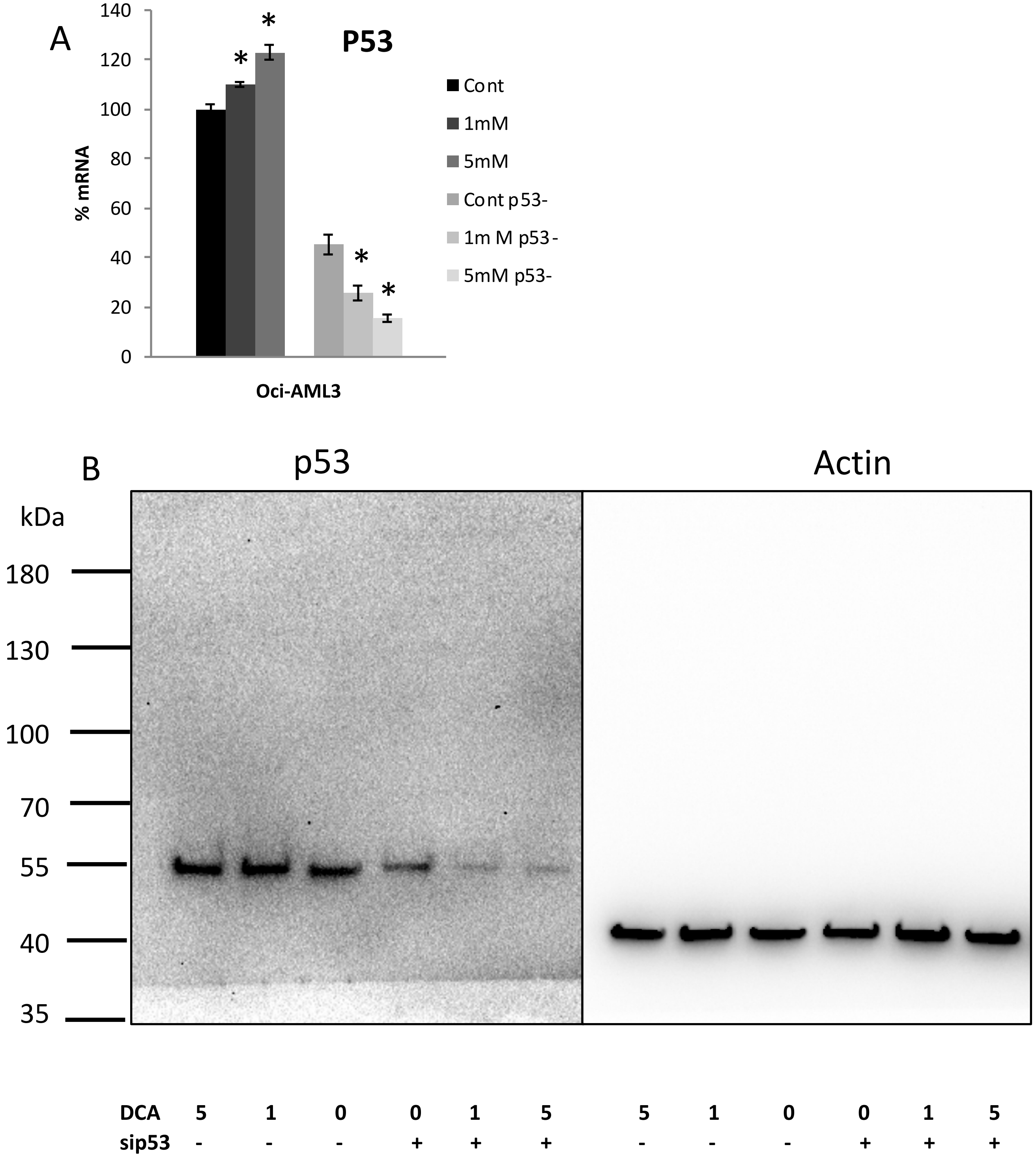

Supplemental Figure 5. Small interfering RNA (siRNA) for p53 efficiently decrease wt p53 levels. OCI-AML3 cells were transfected with siRNA for p53 (sip53) or a control siRNA and 24h later were treated with 1 and 5 mM DCA for 3 days. Protein (B) and mRNA (A) levels were quantified as described in Material and Methods. A) Bar graph denote the % of mRNA compared to control cells and represent means ± SD of 3 independent experiments; * $p<0.05$, student t-test compare to control cells. B) Cells were treated as in (A) and p53 and actin proteins were analyzed by western blot.
